# Integrating and developing computational thinking in higher education using micro:bit: examining the influence on pre-service teachers’ self-perceived content knowledge, attitude, and emotion toward further K-12 mathematics education

**DOI:** 10.3389/fpsyg.2026.1742317

**Published:** 2026-01-29

**Authors:** Jin Su Jeong, David González-Gómez

**Affiliations:** Department of Science and Mathematics Education, Teacher Training College, University of Extremadura, Cáceres, Spain

**Keywords:** mathematics education, computational thinking, micro:bit, content knowledge, attitude, emotion, pre-service teacher, K-12 education

## Abstract

Computational thinking (CT) is widely recognized as a critical 21st-century skill and an essential component of curricula in higher education institutions (HEIs) worldwide. Developing this competence is particularly important for teachers to effectively support K-12 students. However, research on effective strategies for teaching CT within mathematics education remains scarce. This study addresses this gap by investigating the integration and development of CT content knowledge through educational activities incorporating micro:bit technology for pre-service mathematics teachers. In addition, the study examines attitudes and emotions associated with the learning process. The intervention consisted of a six-step sequence: Introducing CT concepts through divisibility content, progressing to visual block programming and Python coding, and culminating in the construction of a physical calculator to identify factors and divisors. A sample of 228 pre-service teachers, initially underprepared to teach CT concepts, participated in various courses employing a pre-experimental design. The influence of the instructional approach on participants’ self-perceived CT content knowledge, attitudes, and emotions was assessed, along with the homogeneity of these effects across the sample. Findings revealed significant increases in self-perceived CT content knowledge and positive attitudes following the intervention, while two negative emotions such as frustration (activating) and boredom (deactivating) persisted regardless of individual characteristics. Thus, the results of this study indicate that the implementation of the micro:bit activity revealed significant improvements in CT components. These results provide further support for the internal consistency and preliminary construct validity of the proposed measurement instruments and underscore the potential benefits of integrating CT into pre-service teacher education in HEIs to enhance future K-12 mathematics instruction.

## 1 Introduction

Computational thinking (CT) is a problem-solving approach that uses computer science concepts such as decomposition, pattern recognition, abstraction, and algorithm design, and is widely acknowledged as a fundamental 21st-century skill (Grover, 2022; Newton et al., 2022; Wing, 2006) in an increasingly digitalized society (Barr and Stephenson, 2011). Technological advancements have transformed the structure of daily life, influencing domains far beyond computer science (Barr and Stephenson, 2011; Deguchi et al., 2020). The importance of CT competencies is reflected in both national and international policy documents, notably within the European Digital Competence Framework for citizens, where CT is identified as a core objective for primary and secondary education (Bocconi et al., 2022; Grover and Pea, 2013; Herrmann et al., 2020).

In particular, the integration of CT into K-12 educational contexts as a practical tool to achieve these objectives has received growing attention (Grover and Pea, 2013; Jocius et al., 2021; Sırakaya et al., 2020). Further research is needed to clarify how CT can foster mathematical thinking and learning in K-12 education, and vice versa (Hickmott et al., 2018; Ye et al., 2023). At the same time, teachers must be adequately prepared to cultivate CT competencies among their students, given the increasing emphasis on developing computationally competent learners and formally incorporating CT into curricula (Carpenter et al., 1989; Miller, 2019; Pei et al., 2018; Shumway et al., 2021). To meet these curricular demands, teachers require a solid foundation in CT content knowledge (Angeli et al., 2016; Caskurlu et al., 2022; Computer Science Teachers Association The International Society for Technology in Education, 2011). Delivering high-quality instruction necessitates a deep understanding of the resources and concepts to be taught, as this directly influences student learning outcomes, particularly in mathematics education, which is widely adopted in K-12 contexts (Baumert and Kunter, 2013a; Cui and Ng, 2021; Putnam et al., 1992).

To effectively integrate and develop computational thinking (CT) within K-12 mathematics education, selecting an appropriate computing device is essential (Barcelos et al., 2018; Hickmott et al., 2018; Lee et al., 2023). Physical computing devices offer opportunities to enhance mathematical learning by making it more engaging, accessible, and conducive to creativity and collaboration (Kalelioglu and Sentance, 2019; Pei et al., 2018; Shumway et al., 2021). These devices support a variety of CT-related activities, including programming tasks and instructional design, which can be embedded into mathematics learning (De Chenne and Lockwood, 2022; Kotsopoulos et al., 2017). Existing studies highlight pedagogical frameworks such as unplugged activities, tinkering, problem formulation, remixing designs, problem-solving, number theory, and mathematical modeling as effective approaches for integrating CT into K-12 mathematics education (Bråting and Kilhamn, 2021; Miller, 2019; Ng and Cui, 2021). Importantly, applying CT in mathematics can deepen conceptual understanding due to the reciprocal relationship between computer science and mathematical models, while disciplinary mathematical knowledge simultaneously strengthens CT skills (Aytekin and Topçu, 2024; Pei et al., 2018; Przybylla and Romeike, 2014).

Among physical computing devices, micro:bit stands out for its robust features and suitability for educational contexts (Ball et al., 2016). According to experienced teachers, micro:bit is user-friendly, affordable, and requires minimal preparation, addressing limitations found in earlier devices (Sentance et al., 2017; Kalelioglu and Sentance, 2019). Its interactive feedback mechanisms make learning more engaging, motivating, and stimulating for students (Lu et al., 2022; Sentance et al., 2017). Furthermore, as students become familiar with its components, they can leverage micro:bit to create increasingly complex and innovative projects (Fagin et al., 2001; Kalelioglu and Sentance, 2019).

CT abilities in K-12 mathematics education, such as problem-solving, remain relatively low due to several contributing factors (Nuraisa et al., 2021; Supiarmo et al., 2022; Wardani et al., 2021). In this context, teachers’ CT content knowledge within higher education institutions (HEIs) is still insufficiently developed, despite growing research interest in this area (Kong et al., 2023; Tang et al., 2020; Yadav et al., 2016). Baumert and Kunter (2013b) emphasize that content knowledge constitutes a core teaching competence, requiring teachers to possess a deep understanding of the subject matter to be taught (Mishra and Koehler, 2006; Shulman, 1986), as well as related pedagogical and domain-specific skills (Baumert and Kunter, 2013b; Depaepe et al., 2013; Star, 2005). Moreover, it is essential to examine the relationship between attitudes and emotions and CT content knowledge in K-12 mathematics education (Bieleke et al., 2023; González-Gómez et al., 2022; Lu et al., 2022). Previous research indicates that attitudes influence CT content knowledge and learning outcomes for both students and pre-service teachers (Cutumisu et al., 2021; Jeong and González-Gómez, 2025a; Sun et al., 2021). Learners’ attitudes and emotions toward activities involving micro:bit technology are closely linked to mathematics education (Humble, 2022; Voštinár and Knežník, 2020; Wadaani, 2023). Emotions, in particular, have gained prominence due to their significant impact on learning outcomes in CT (Bieleke et al., 2023; Herrero-Álvarez et al., 2023; Jeong et al., 2019). Mathematics education is a critical subject globally and is frequently associated with a wide range of emotions, both positive and negative, making it an important area of investigation (Frenzel et al., 2007; Pekrun et al., 2007; Schukajlow et al., 2017).

Thus, this study aims to integrate and develop CT through a micro:bit-based activity (CT-micro:bit) by examining pre-service teachers’ self-perceived CT content knowledge (measured via decomposition, pattern recognition, abstraction, and algorithm design), attitudes (measured via creativity, problem solving, algorithmic thinking, cooperation, and critical thinking), and emotions (measured via emotional stability, self-motivation, and emotional relations) in preparation for future K–12 mathematics teaching. The research is guided by the following questions:

1. Can the CT-micro:bit activity improve confidence in content knowledge among pre-service teachers in HEIs for further K-12 mathematics education?
2. Can the CT-micro:bit activity influence the attitude for pre-service teachers in HEIs for further K-12 mathematics education?
3. Can the CT-micro:bit activity affect the emotion (positive, negative, activating, and deactivating construct) for pre-service teachers in HEIs for further K-12 mathematics education?

The research results could be considered as a further suggestion, how pre-service teachers could improve in further K-12 mathematics learning to enable the development of self-perceived content knowledge, attitude, and emotion of CT through the activity of physical computing device like micro:bit.

## 2 Materials and methods

**Figure 1:**
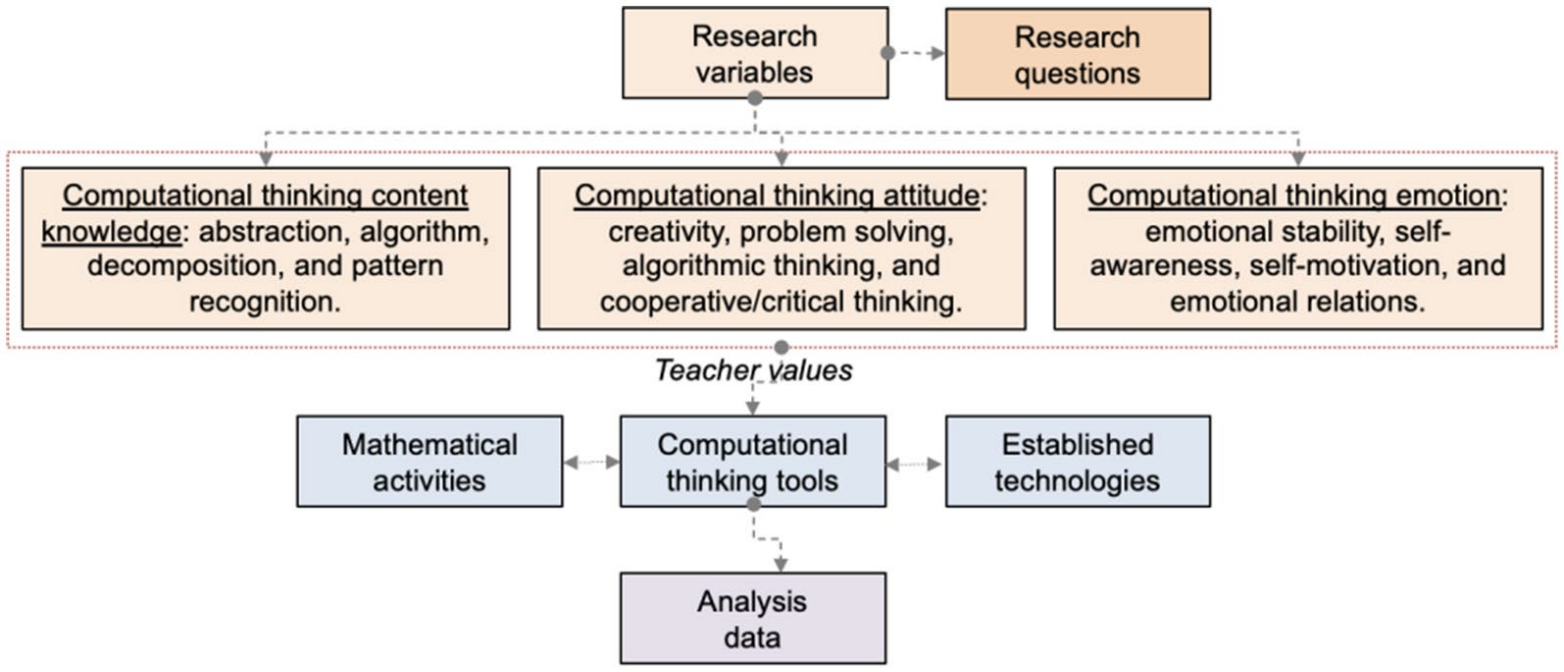
Research scheme to examine the variables proposed (Zaho and Frank, 2003).

This study investigates the self-perceived content knowledge, attitudes, and emotions associated with CT among pre-service teachers in HEIs through micro:bit-based activities. The research aims to potentially enhance preparation for K-12 mathematics education. A pre-experimental design was adopted, employing quantitative descriptive data to compare pre- and post-test results across various academic courses (Maxwell et al., 2017). Additionally, a correlational design was used to identify variables that may predict outcomes (Creswell and Creswell, 2018). The conceptual framework is illustrated in Figure 1.

### 2.1 Course description

The course selected and implemented for this research is a compulsory introductory subject titled Mathematics and its Didactics. It carries 6 credits and comprises a total of 150 h, aimed at developing the course competencies. The course forms part of the core curriculum of the Primary Education degree and employs a range of didactic approaches to prepare pre-service teachers to teach mathematics in primary schools.

The course content spans six chapters, covering definitions, characterizations, themes, theories, and principles relevant to mathematics education, with a particular emphasis on arithmetic concepts and instruction. For the purposes of this study, Chapter 4, Divisibility, was selected. This chapter is divided into four sub-units, each taught over 1 week: (1) prime and composite numbers; (2) divisors of a number; (3) greatest common divisor and least common multiple; and (4) divisibility criteria. The overarching goal of this course is to equip pre-service teachers with the necessary content knowledge, competencies, teaching methodologies, and research skills to effectively educate future K-12 students on mathematics education. This is especially important given that many pre-service teachers are not adequately prepared to teach these mathematical concepts with confidence and clarity.

### 2.2 Pre-service teachers’ participants

The participants in this study were 228 pre-service teachers enrolled in the Primary Education degree program at the authors’ university, with 110 and 118 students participating in various academic years, respectively. The study was conducted during the second semester of both academic years. Table 1 presents the main demographic characteristics of the participants, collected via an online quantitative survey that included an informed consent form. In the first collection, 110 pre-service teachers participated, with an average pre-grade score of 7.12 and a mean age of 19.93 years. In the second collection course, 118 pre-service teachers participated, with an average pre-grade score of 7.29 and a mean age of 20.44 years. Regarding gender distribution, the first collection included 76 female and 34 male participants, while the second collection comprised 74 female and 46 male participants. All participants were engaged in the same learning environment. Finally, in terms of university access and educational background, the majority of pre-service teachers came from high school programs with a focus on social sciences.

**Table 1:** Pre-service teachers’ demographic information during academic courses.

**Participant**		**Pre-grade** **(Max. 10)**	**Age**	**Gender**		**University access**			**Background**		
				**Female**	**Male**	**High school**	**Professional school**	**Test over age 25**	**Social science**	**Science**	**Technology**
First	110	7.12	19.93	76	34	100	10	0	66	22	22
Second	118	7.29	20.44	74	46	100	18	0	78	30	10

Throughout the various academic years during which this research was conducted, the same professor was responsible for teaching the course content and instructional methodologies, in accordance with the official curriculum established by the authors’ university.

### 2.3 Research instrument and procedure

**Figure 2:**
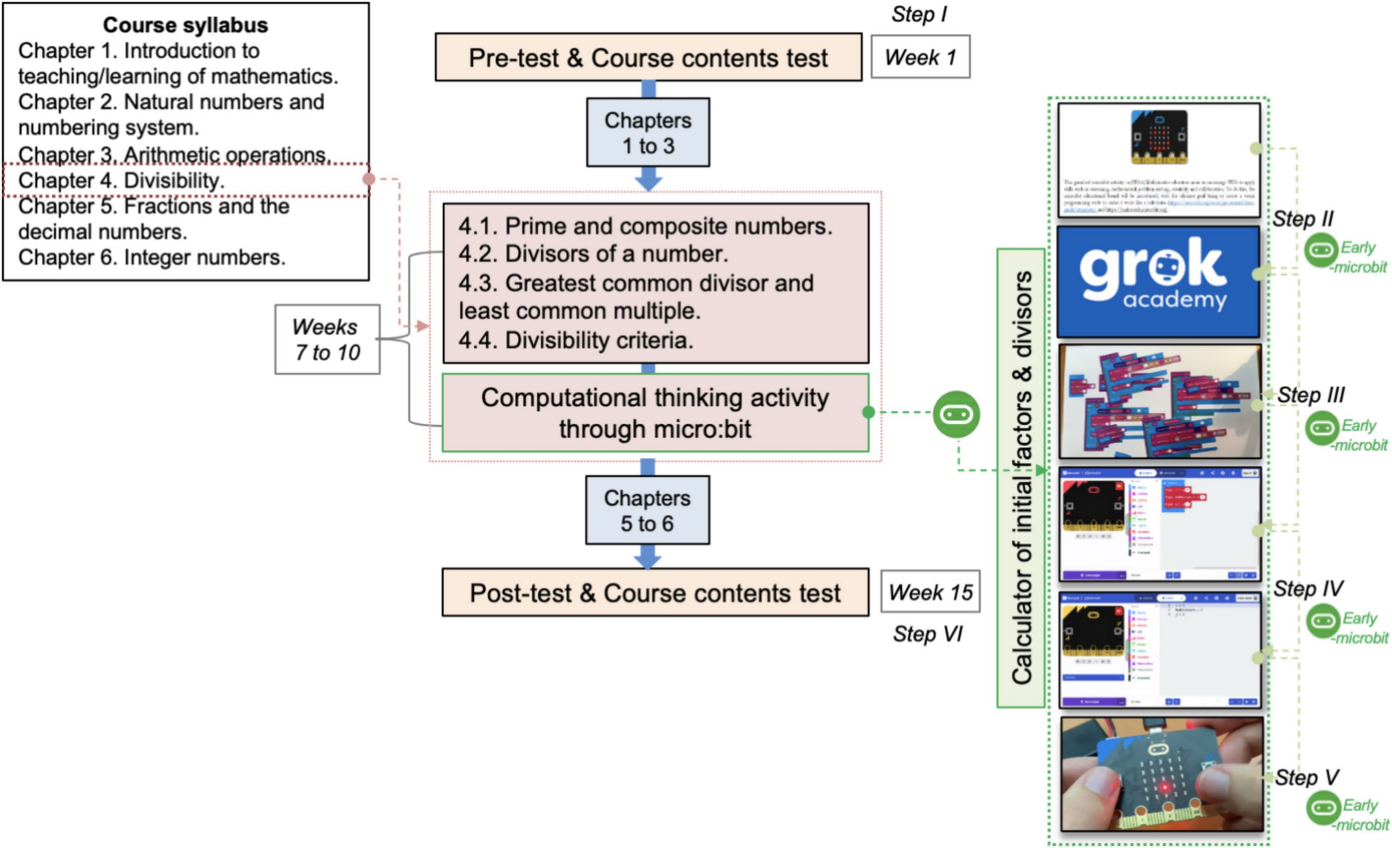
General research procedure to integrate and develop computational thinking through micro:bit activity of university mathematics course.

To integrate and develop computational thinking through a micro:bit-based activity in K-12 mathematics education for pre-service teachers, Figure 2 presents an overview of the research instruments and procedures. In alignment with the university’s official syllabus, the intervention was implemented in Chapter 4 (Divisibility) of the course.

**Figure 3:**
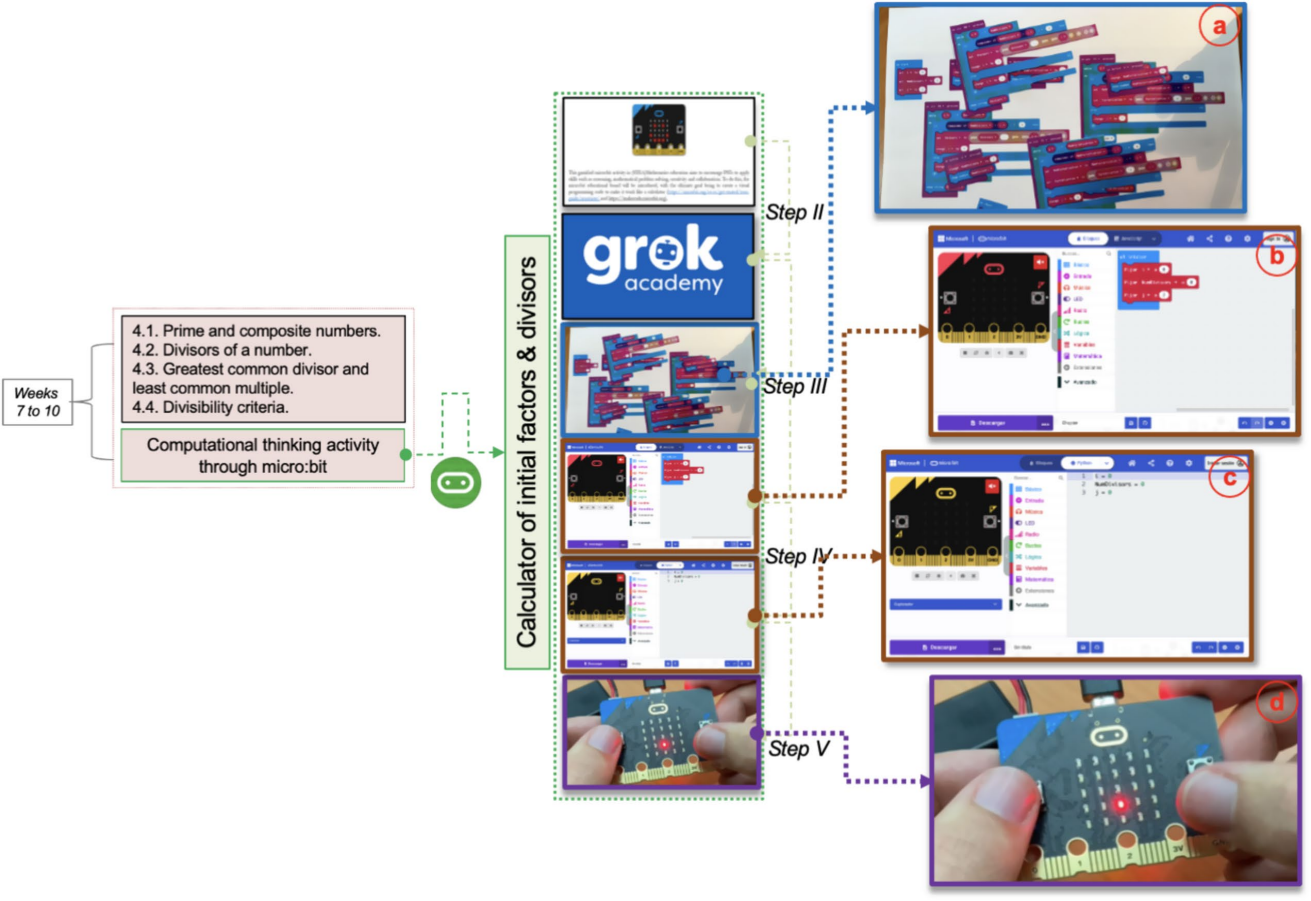
Steps III to V of micro:bit activity of university mathematics course.

The pre-experimental study was conducted over 4 weeks, corresponding to the duration of this chapter, and involved a six-step instructional activity. The class sessions totaled 4 h per week, delivered in two sessions. Step I (Week 1): Pre-service teachers completed an anonymous homogeneity assessment focused on subject content, along with an additional anonymous questionnaire evaluating their self-perceived CT knowledge, attitudes, and emotional responses related to mathematics education. Step II: The general educational micro:bit activity was introduced, aimed at developing a calculator for identifying prime factors and divisors, aligned with the content of Chapter 4. This step included an overview of the activity process and implementation, supplemented by a course on the Grok Academy platform. This course supported the development of visual block-based and Python programming skills using micro:bit, enabling pre-service teachers to engage with CT in an accessible way. Step III: Participants engaged in an unplugged activity using manipulative visual black materials (see Figure 3a). This step served as a transitional phase, gradually introducing coding concepts without the use of digital technology. Pre-service teachers were required to design program sequences manually, fostering foundational algorithmic thinking. Step IV: In the first plugged activity, participants used the Microsoft MakeCode web platform to program their calculators. Initially, they worked with visual block coding and subsequently transitioned to Python (see Figures 3b,c). This step built directly on the knowledge and skills acquired in Steps II and III. Step V: Pre-service teachers constructed a physical calculator using the micro:bit device (see Figure 3d). This hands-on activity allowed them to explore the micro:bit’s structure and functionality, including visual feedback and input management features. The device enabled execution of various operations, reinforcing the connection between abstract coding concepts and tangible outcomes. Step VI (Week 15): The final step mirrored Step I, with participants completing a post-intervention anonymous questionnaire assessing their computational thinking knowledge, attitudes, and emotional responses toward mathematics education. Throughout Steps II to V, participants earned “early-microbit insignias” as part of a communal challenge. These insignias served to discourage shortcuts and ensure full engagement with the designed instructional sequence (Baker et al., 2008; Hew et al., 2016). Although the instructor had prior training in computational thinking, educational tools, and programming languages, the research design was further supported through a research-practice partnership with university experts (Coburn and Penuel, 2016).

### 2.4 Data instrument and collection

The data were collected through quantitative pre- and post-tests administered online using a 5-point Likert-type scale. A computational thinking survey was developed to evaluate pre-service teachers’ pre-perceived CT content knowledge, attitudes, and emotions toward mathematics education, as well as their understanding of computational thinking, both prior to and following the implementation of a CT-micro:bit activity module. The survey comprised three instruments encompassing a total of 16 items, with response options ranging from “strongly disagree” to “strongly agree” (see Table 2). These instruments were adapted from previously validated studies addressing three proposed constructs within the context of computational thinking. Items measuring content knowledge were derived from Krauss et al. (2008) and Jüttner et al. (2013). Attitudinal items were adapted from Cutumisu et al. (2021) and Sun et al. (2021). Finally, items assessing emotional dimension were adapted from Jeong et al. (2019), Díaz and Flores (2001), and Pekrun et al. (2007).

**Table 2:** Questionnaries of pre-perceived computational thinking content knowledge, attitude, and emotion of pre-service teachers with micro:bit activity toward mathematics learnings.

#	Type		Question		Indicator	Resource
1	Pre-perceived computational thinking content knowledge		I am able to explain overall pattern of similarities/dissimilarities that have been discovered.		Abstraction	Krauss et al. (2008) and Jüttner et al. (2013)
2			I am able to designate the logical phases utilized to make a solution.		Algorithm	
3			I am able to identify information from problems and/or in the form of questions from problems.		Decomposition	
4			I am able to recognize similarities/dissimilarities of patterns/characteristics in problem solving for building resolutions.		Pattern recognition	
1	Computational thinking attitude		I believe that I can resolve the problems that probable to arise when I confront in a new setting.		Creativity	Cutumisu et al. (2021) and Sun et al. (2021)
2			I believe that I can employ the resolution through the means I plan individually and progressively.		Problem solving	
3			I believe that I can find out the relationship between concepts and symbols.		Algorithmic thinking	
4			I believe that I can organize proposals regarding to the resolution of the complicated problems.		Cooperative/ critical thinking	
1	Computational thinking emotion	Positive	Joy	Activating	Emotional stability, self-awareness, self-motivation, and emotional relations	Díaz and Flores (2001), Jeong et al. (2019) and Pekrun et al. (2007)
2			Enthusiasm			
3			Satisfaction	Deactivating		
4			Confidence			
5		Negative	Nervousness	Activating		
6			Frustration			
7			Boredom	Deactivating		
8			Disappointment			

The questionnaire and research protocol received approval from the university’s Bioethics and Biosecurity Committee (Reference No. 94/2018 and 200/2024) to ensure compliance with ethical standards in collecting computational thinking data from pre-service teachers prior to initiating the study. In alignment with the research-practice partnership approach (Coburn and Penuel, 2016), different university professors specializing in mathematics education were engaged to examine and validate the proposed questionnaire.

### 2.5 Data analysis

Prior to conducting the data analysis, the homogeneity of participants was evaluated. This evaluation involved analyzing scores from a pre-contents test to ascertain whether the data conformed to a normal distribution and demonstrated homogeneity. The Shapiro–Wilk test indicated that the data were normally distributed (*p =* 0.112), while Levene’s results (*p* = 0.387) confirmed that the pre-contents test scores were homogeneous across all participants. Then, the internal consistency of each instrument was assessed using Cronbach’s alpha, yielding values of 0.870, 0.838, and 0.907 for the “pre-perceived computational thinking content knowledge” (CTK), “computational thinking attitude” (CTA), and “computational thinking emotion” (CTE) scales, respectively, and, therefore, could be considered acceptable (Cho and Kim, 2015). To identify potential latent factors influencing the observed data, an exploratory factor analysis (EFA) was performed. Bartlett’s test of sphericity was significant for CTK (*p* < 0.001), CTA (*p* < 0.001), and CTE (*p* < 0.001). Additionally, the Kaiser–Meyer–Olkin (KMO)‘s measure of sampling adequacy (MSA) was as well determined being 0.790 for CTK, 0.811 for CTA, and 0.899 for CTE, indicating good sampling adequacy. Finally, the number of factors was calculated using the minimum residual extraction method with an *oblimin* rotation. Table 3 presents the factor loadings for each instrument (CTK, CTA, and CTE). These results enable the calculation of a global score by equally weighing the scores from the included variables within each section (G-CTK, G-CTA, and G-CTE). For the G-CTE, the negative emotions 2 and 3 (frustration and boredom) were excluded based on the EFA results.

**Table 3:** Factor loadings and factors summary statistics for the “pre-perceived computational thinking content knowledge” (CTK), “computational thinking attitudes” (CTA), and “computational thinking emotions” (CTE).

Pre-perceived computational thinking content knowledge				
Variable	Factor 1	Uniqueness		
CTK_1	0.675	0.5441		
CTK_2	0.858	0.2640		
CTK_3	0.699	0.5121		
CTK_4	0.965	0.0691		
	Factor	SS Loadings	% of Variance	Cumulative %
Factor statistics	1	2.61	65.3	65.3

Computational thinking attitude				
Variable	Factor 1	Uniqueness		
CTA_1	0.711	0.494		
CTA_2	0.868	0.246		
CTA_3	0.863	0.254		
CTA_4	0.664	0.560		
	Factor	SS Loadings	% of Variance	Cumulative %
Factor statistics	1	2.45	61.1	61.1

Computational thinking emotion				
Variable	Factor 1	Factor 2	Uniqueness	
CTEP_1	0.795		0.342	
CTEP_2	0.723		0.430	
CTEP_3	0.843		0.291	
CTEP_4	0.899		0.196	
CTEN_1	0.689		0.518	
CTEN_2		0.389	0.849	
CTEN_3		0.398	0.842	
CTEN_4	0.797		0.368	
	Factor	SS Loadings	% of Variance	Cumulative %
Factor statistics	1	3.784	47.30	47.3
	2	0.382	4.78	52.1

After refining the instrument and computing new variables, the influence of the proposed methodology was assessed by comparing the variables before and after the intervention (pre- and post-test comparison). To do this, the Kolmogorov–Smirnov test was first applied to check whether the dependent variables were normally distributed (for G-CTK, G-CTA, and G-CTE). Since the data were normally distributed (*p* > 0.05), the Student’s *t*-test for paired samples was used to compare pre- and post-intervention scores. Furthermore, a one-way ANCOVA was conducted to examine the influence of the instruction methodology on G-CTK, G-CTA, and G-CTE whilst controlling for covariates such as students’ grade, gender, age, and educational background. Before conducting the ANCOVA test, Levene’s test and normality checks were carried out to check that all assumptions were met. Finally, Spearman Correlation was used to explore relationships between variables before and after the intervention. To identify the strength and direction of association between variables, a partial plot was used. The Jamovi project (2025) software was used for all statistical calculations.

## 3 Results and discussion

**Figure 4:**
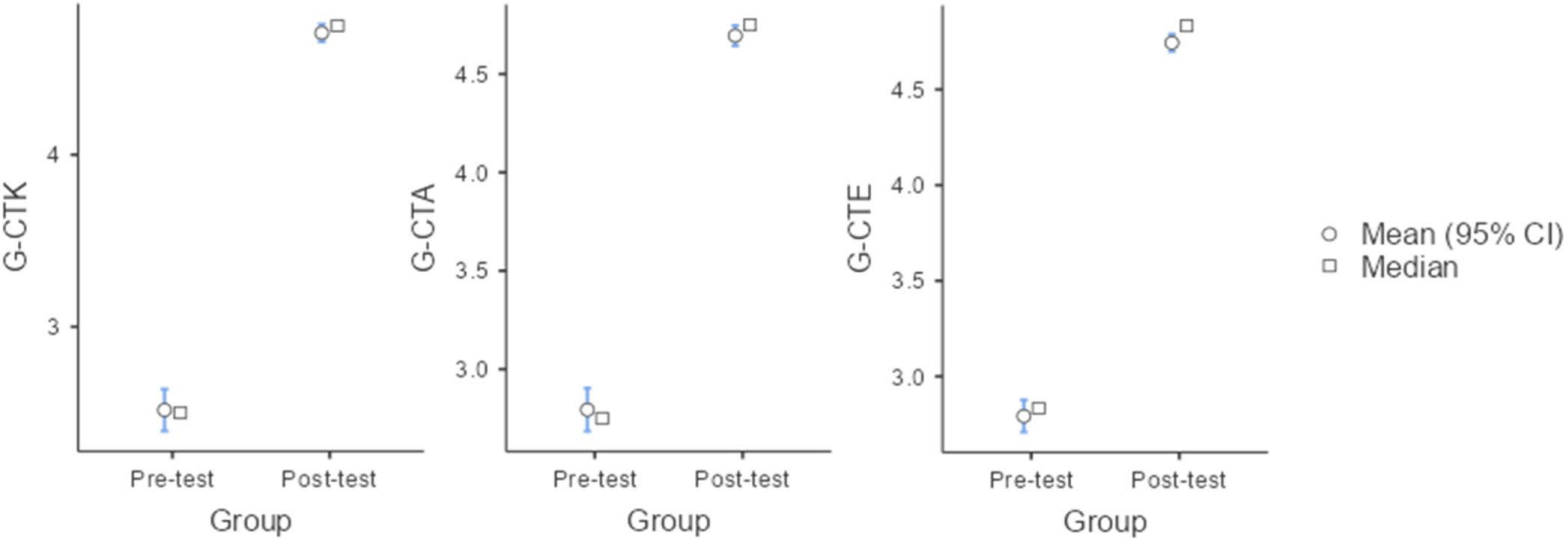
Mean values of the G-CTK, G-CTA, and G-CTE before and after the intervention (pre- and post-test results) with the associated 95% confidence intervals.

To assess whether the implementation of an instruction methodology based on the use of micro:bit to integrate and develop the computational thinking on mathematics educations of pre-service teachers had a significant influence on pre-service teachers on the dependent variables, the mean values of the G-CTK, G-CTA, and G-CTE were compared before and after the intervention. For G-CTK, the mean score before the intervention was 2.50 (std dev = 0.66), which increased to 4.71 (std dev = 0.28) after the intervention. The Student’s *t*-test for paired samples indicated that this difference was statistically significant (*t*(226) = 32.4, *p* < 0.001, *CI*_95_ = [2.06, 2.33], *d* = 4.39). This result indicates a statistically significant increase in self-reported confidence regarding computational thinking content knowledge from pre- to post-test instructional intervention. Similar results were observed for G-CTA, where the mean score increased from 2.79 (std dev = 0.60) before the intervention to 4.70 (std dev = 0.28) after it. The *t*-test confirmed that this difference was also statistically significant (*t*(226) = 30.8, *p* < 0.001, CI*_95_* = [1.78, 2.02], *d* = 4.08). Finally, for G-CTE, the mean score rose from 2.70 (std dev = 0.46) prior to instruction to 4.74 (std dev = 0.25) post-instruction (*t*(226) = 40.19, *p* < 0.001, CI*_95_* = [1.86, 2.04], *d* = 5.32). This difference was also statistically significant. Figure 4 represents the evolution of these variables before and after the intervention. In all cases, the effect size, measured as Cohen’s *d*, denotes that large pre-post differences were observed across all the studied variables, although these should be interpreted cautiously given the pre-experimental design. The large effect size values may indicate the presence of a ceiling effect, potentially introducing a limitation to the overall measurements. However, it is important to note that the effect sizes calculated for each individual variable fall within the conventional thresholds for Cohen’s *d.*

**Figure 5:**
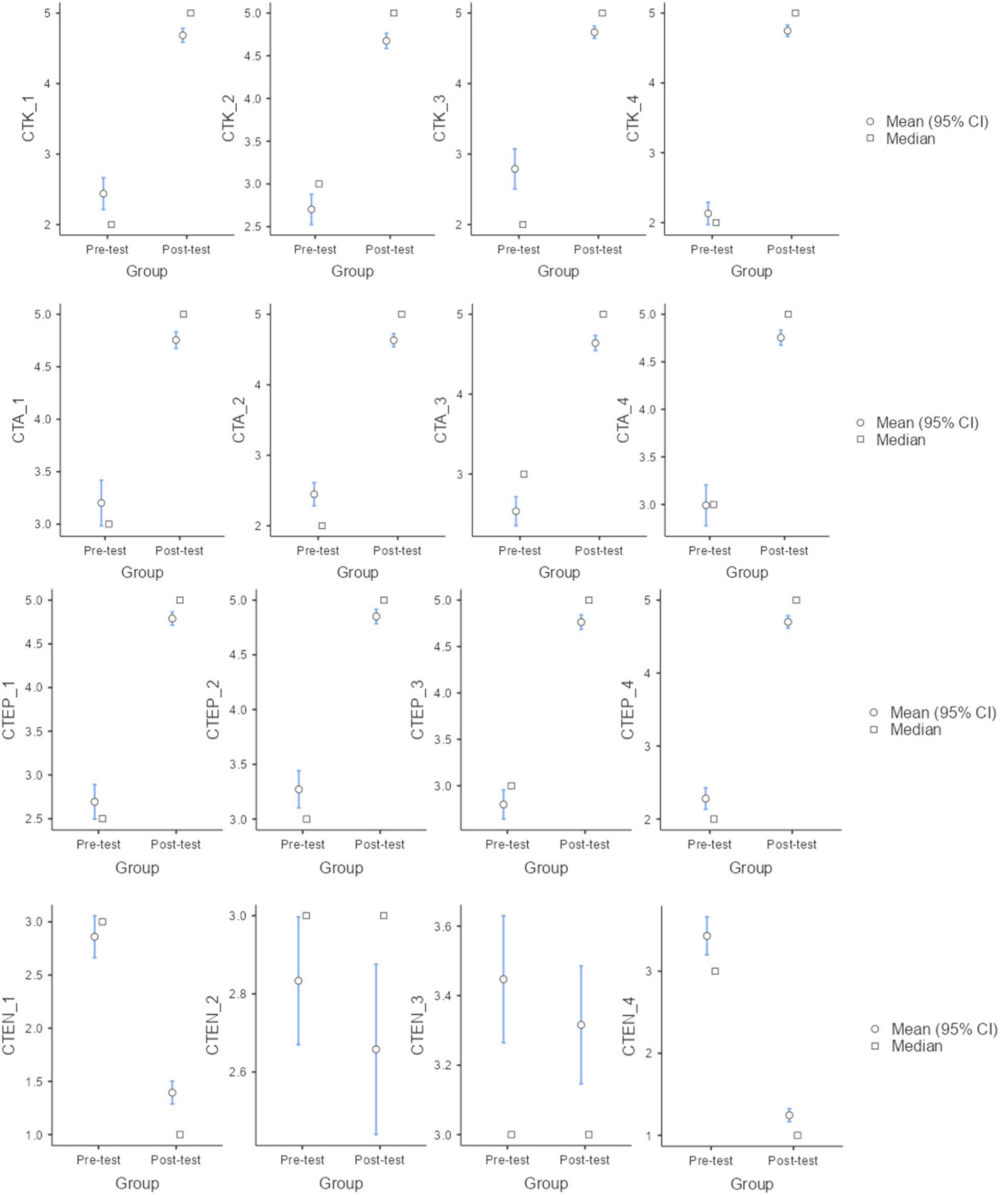
Evolution of the mean values of the CTK, CTA, and CTE set of variables before and after the intervention (pre- and post-test results) with the associated 95% confidence intervals.

A one-way ANCOVA was conducted to assess the influence of the instructional methodology on the students’ G-CTK, G-CTA, and G-CTE whilst controlling students’ grade, gender, age, and educational background. ANCOVA results indicated that there was a significant difference in mean values of G-CTK [*F*(1,221) = 1054.4, *p* < 0.001], G-CTA [F(1,221) = 948, *p* < 0.001], and G-CTE [F(1,221) = 1609.8, *p* < 0.001] before and after the intervention, respectively. However, the results of the ANCOVA test also indicated that students’ grades, gender, age, and educational background did not influence the dependent variables assessed in this study. On the other hand, to have a better understanding of how each individual score sifted within the intervention, Figure 5 represents the mean values of each individual item before and after the intervention. As shown in Figure 5, all items in the “pre-perceived computational thinking content knowledge” domain exhibited significant pre–post improvement (*p* < 0.001). The largest mean differences were observed for abstraction (Item 1; ΔM = 2.25) and pattern recognition (Item 4; ΔM = 2.65). Comparable effects were found in the “computational thinking attitude” domain (*p* < 0.001), with problem solving (Item 2; ΔM = 2.18) and algorithmic thinking (Item 3; ΔM = 2.11) showing the greatest gains. Regarding the “computational thinking emotion,” in the case of the positive one, a significant positive influence was observed (*p* < 0.001). On the other hand, for the negative emotions the mean values of observed in “Nervousness” (CTEN_1) and “Disappointment” (CTEN_4) significantly decreased after the intervention (*p* < 0.001), while “Frustration” (CTEN_2) and “Boredom” (CTEN_3) did not experience any change during the intervention (*p* = 0.209 and *p* = 0.302, respectively).

Considering the activating/deactivating classification of emotions (Pekrun et al., 2007), results indicate that positive activating emotions (CTEP_1 and CTEP_2) significantly increased after the intervention, and that could be related to an enhancement of learning and performance (Pekrun, 2010). On the other hand, the positive deactivating emotions (CTEP_3 and CTEP_4) increased after the intervention. Although limited research is available on their specific effects, fostering these emotions could potentially reduce cognitive stress and might offer a temporary respite from intense cognitive engagement (Dreisbach and Goschke, 2004). Regarding the negative activating emotions (CTEN_1 and CTEN_2), a significant decrease was observed in CTEN_1 (nervousness) while no difference was observed in CTEN_2 (frustration). The negative activating emotions, in general terms, affect students’ motivation and reduce self-regulating learning (Heckhausen, 1991). In the case of the negative deactivating emotions (CTEN_3 and CTEN_4), no significant difference was observed for CTEN_3 (boredom) while a significant decreased was observed for CTEN_4 (disappointment). Negative deactivating emotions negatively influence students’ learning process and outcomes, therefore the instruction methodology implemented might mitigate these effects. The large Cohen’s *d* values for the overall scores derived from the EFA results may indicate a potential ceiling effect, which could constrain the interpretation of these scores when assessing the intervention’s influence. At the item level, pre–post Cohen’s *d* values ranged from *d* = 0.67 to *d* = 0.88, all remaining below 0.90. This item-level analysis strengthens the validity of the study by complementing the interpretation of the global results, while factors such as the use of self-report measures and the absence of a control group may be also acknowledged.

Finally, Table 4 represents the plot of the Spearman correlations and network plot for the validated subscale scores (G-CTK, G-CTA, and G-CTE) and the two CTE factors (frustration and boredom) identified through exploratory factor analysis before and after the intervention. In this plot, the color of the lines denotes the positive (green color) or negative (red color) relationship between variables, and the line thickness the strength of this relation.

**Table 4:** Partial correlation and network plot for the validated subscale scores (G-CTK, G-CTA, G-CTE) and the two CTE factors identified through exploratory factor analysis.

Network Plot		Pre-test group			Post-test group		
		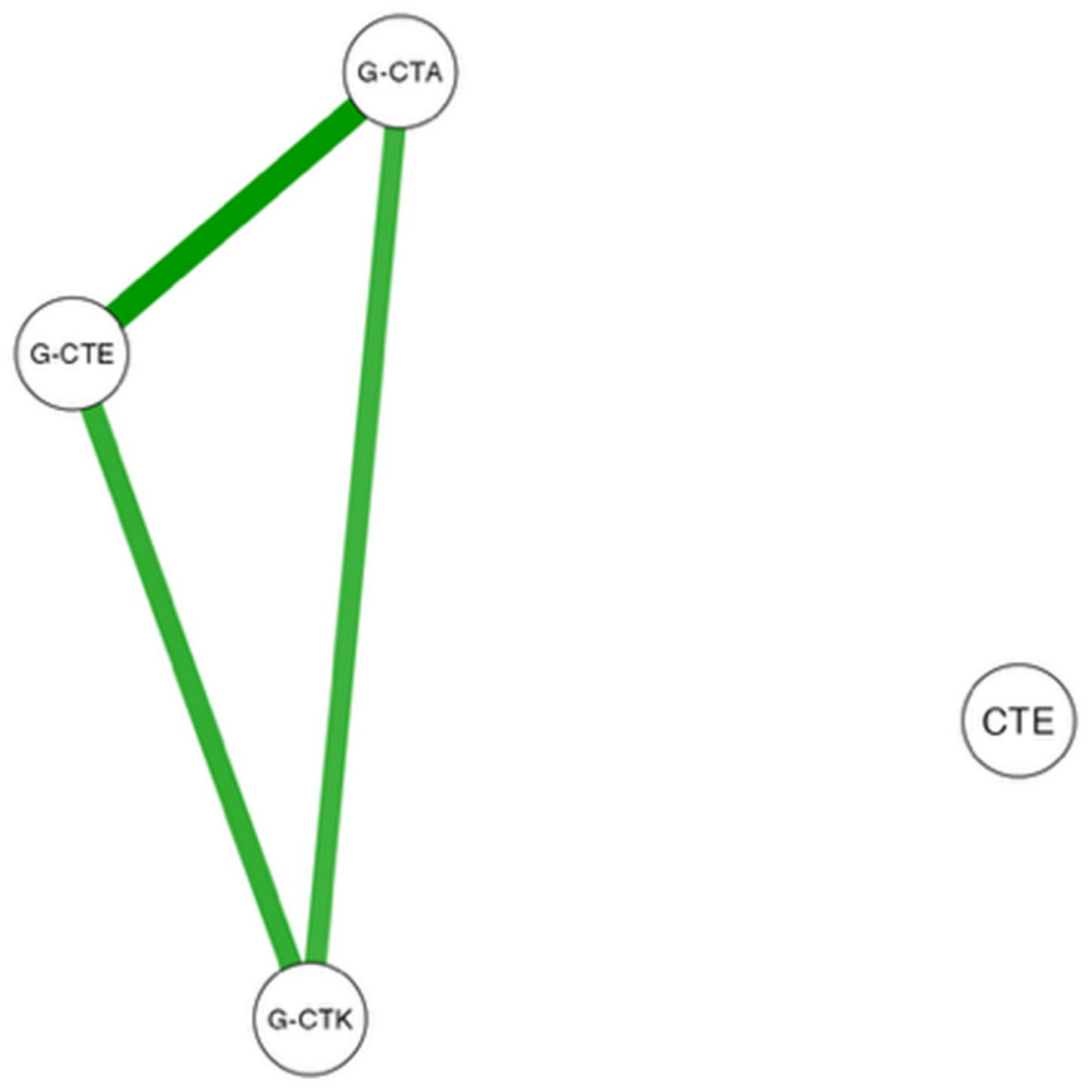			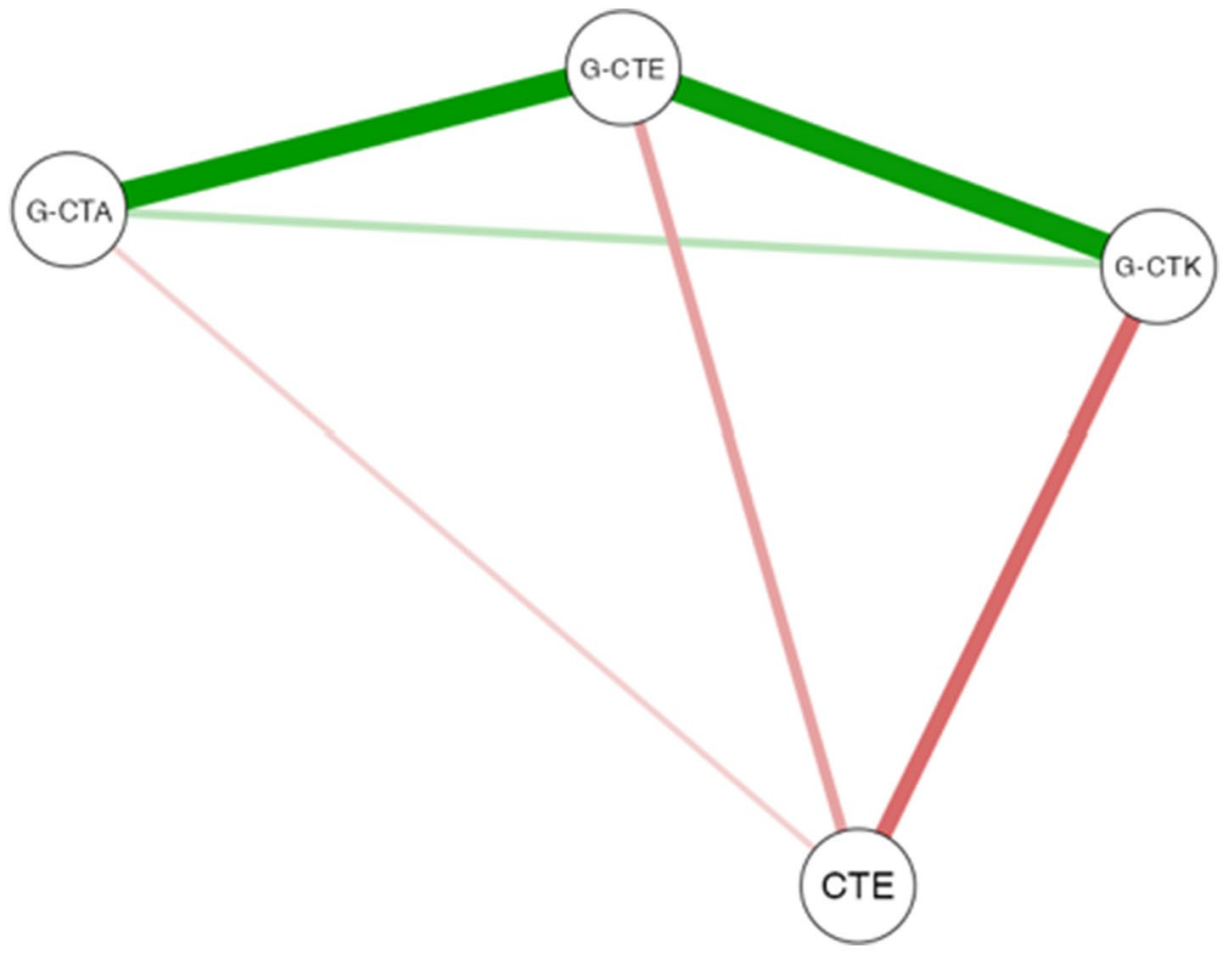		
Sub-scale Scores		G-CTK	G-CTA	G-CTE	G-CTK	G-CTA	G-CTE
G-CTA	rs	0.839***			0.383***		
G-CTE	rs	0.835***	0.857***		0.570***	0.537***	
CTE	rs	−0.087	−0.049	−0.050	−0.345***	−0.300**	−0.331***

The significance levels of the correlations are indicated as *p < 0.05, **p < 0.01, and ***p < 0.001.

According to these results, prior to the intervention, G-CTK, G-CTA, and G-CTE exhibited significant positive correlations with each other (*r*_s_ > 0.8 and *p* < 0.001 in all cases), while no correlation was observed with the two CTE factors (frustration and boredom). After the intervention, significant correlations among G-CTK, G-CTA, and G-CTE persisted (*r*_s_ ranging between 0.4 to 0.6 and *p* < 0.001, as indicated in Table 4). Notably, a significant negative correlation also emerged between the CTE constructs (frustration and boredom) and the three subscales (G-CTK, G-CTA, and G-CTE) (*r*_s_ < −0.3 and *p* < 0.001 in all cases), with the strongest negative association observed between CTE and G-CTK. This finding may provide insight into the effectiveness of the intervention in enhancing pre-perceived CT content knowledge.

Based on the results analyzed through various approaches, the study addressed the first research question raised for this study (Q1). Tang et al. (2020), along with other researchers (Kong et al., 2023; Yadav et al., 2016), indicated that the teachers’ content knowledge in this field of HEIs is not well-established for K-12 mathematics education. However, the findings revealed significant pre-post differences following the implementation of the CT-micro:bit activity sequence. In detail, as it was mentioned before, the mean scores of G-CTK increased 2.25 to be 4.71 (std dev = 0.28) after the intervention and this difference was statistically significant, which suggested a significant improvement. Therefore, these findings align with the previous research emphasizing the importance of developing teachers’ confidence and perceived readiness related to computational thinking (Baumert and Kunter, 2013b; Depaepe et al., 2013; Mishra and Koehler, 2006). Then, Lu et al. (2022) and Bieleke et al. (2023) specified that it is important to know the relationship of attitude and emotion to computational thinking content knowledge in K-12 mathematics education. To answer the second question raised for this study (Q2), Cutumisu et al. (2021) denoted that attitude had a strong relation with the content knowledge of computational thinking, which affected students’ learning achievements in K-12 mathematics education. In detail, the mean of G-CTA increased 1.91 to be 4.70 (std dev = 0.28) after the intervention, and this difference was statistically significant, which suggested a significant improvement. In all cases, the results of the ANCOVA test indicated that students’ grades, gender, age, and educational background did not influence the dependent variables assessed in this study. Therefore, this corresponds to the indications of previous research that, if learners showed positive attitude to a sequence of CT-micro:bit activity, they would more interconnect to K-12 mathematics education (Humble, 2022; Jeong and González-Gómez, 2025b; Sun et al., 2021; Wadaani, 2023). Moreover, to answer the third question raised for this study (Q3), Bieleke et al. (2023) and Herrero-Álvarez et al. (2023) expressed that emotion had a strong relation with the content knowledge of computational thinking, which affected students’ learning achievements in K-12 mathematics education. In detail, the mean of G-CTE increased 2.04 to be 4.74 (std dev = 0.25) after the intervention, and this difference was statistically significant, which suggested a substantial difference. However, “Frustration” (CTEN_2) and “Boredom” (CTEN_3) as negative emotion did not change during the intervention (*p* = 0.209 and *p* = 0.302, respectively). Considering the activating/deactivating classification of emotions (Pekrun et al., 2007), the results showed the same tendency although “Frustration” (CTEN_2) is an activating negative emotion and “Boredom” (CTEN_3) is a deactivating negative emotion regardless of participants’ personal characteristics. Particularly, in a different and challenging learning situation, these emotions are not considered negative since, without them, it would not have the fun component in the learning process (Clauson et al., 2019; Jeong, 2018; Jeong et al., 2016; Sierra and Fernández-Sánchez, 2019). In this context, therefore, this corresponds to the indications of previous research that, if learners showed emotion to a sequence of CT-micro:bit activity, they would more interconnect to K-12 mathematics education (Frenzel et al., 2007; Schukajlow et al., 2017). Thus, the results of this study indicate that the implementation of the micro:bit activity revealed significant pre-post differences in CT components. Specifically, the content knowledge components showed strong gains in concept generalization and recognition of structural regularities. The attitude components indicated a consistent increase in students’ confidence in tackling structured tasks and designing stepwise solutions. Finally, the emotion components suggest that the instructional approach may have mitigated detrimental affect related with computational thinking.

## 4 Conclusion

The proposed study aimed to potentially enhance pre-service teachers’ pre-perceived CT content knowledge, attitudes, and emotions for future K–12 mathematics instruction through the implementation of a CT–micro:bit activity. This intervention was integrated into the mandatory introductory course “Mathematics and its Didactics.” The CT–micro:bit activity followed a six-step sequence aligned with divisibility concepts, introducing participants to visual block-based programming and Python, and culminating in the construction of a physical calculator designed to identify prime factors and divisors.

Regarding the Research Question 1 (Q1), the intervention was associated with statistically significant pre–post improvements in self-perceived CT content knowledge (e.g., gains consistent with abstraction/concept generalization and pattern recognition/structural regularities). These findings indicate that mathematically grounded physical-computing tasks might strengthen pre-service teachers’ confidence in core CT content knowledge relevant to K–12 mathematics teaching. On the other hand, regarding Research Question 2 (Q2), CT attitudes improved significantly, reflecting increased confidence in tackling structured tasks and designing stepwise solutions (e.g., problem solving and algorithmic thinking). These attitudinal gains suggest that the activity fostered productive dispositions toward integrating CT in mathematics pedagogy. Finally, regarding Research Question 3 (Q3), positive emotions increased significantly. Among negative emotions, nervousness (activating) and disappointment (deactivating) decreased significantly, whereas frustration (activating) and boredom (deactivating) showed no detectable change. Interpreted within the activating/deactivating framework, these patterns suggest the instructional approach may have mitigated detrimental affect while enhancing emotions typically associated with learning and performance.

These results suggest that engaging pre-service teachers in physical computing activities may foster more positive self-perceptions of computational thinking content knowledge, attitudes, and emotional engagement, with potential implications for future K–12 mathematics education.

Nevertheless, despite its advantages, incorporating micro:bit activities into educational settings presents challenges, including alignment with curricular objectives and potential disruptions to established lesson schedules. This study also entails limitations that must be acknowledged. First, the pre-experimental design lacks a control group, and the measured effects are based on a single-site context. Second, the instruments relied on self-reported measures, which may introduce bias. Finally, as discussed in the manuscript, the possible existence of a ceiling effect on global scores should be considered. To fully leverage the benefits while mitigating these challenges, a deliberate and systematic approach to integrating CT–micro:bit activities into curricula is essential, incorporating appropriate comparison groups and conducting longitudinal follow-up studies, thereby informing promising educational strategies.

## Data Availability

The original contributions presented in the study are included in the article/supplementary material, further inquiries can be directed to the corresponding authors.
